# Suppressor of Cytokine Signaling-1/STAT1 Regulates Renal Inflammation in Mesangial Proliferative Glomerulonephritis Models

**DOI:** 10.3389/fimmu.2018.01982

**Published:** 2018-08-30

**Authors:** Jiuxu Bai, Lingling Wu, Xiaoniao Chen, Liqiang Wang, Qinggang Li, Yingjie Zhang, Jie Wu, Guangyan Cai, Xiangmei Chen

**Affiliations:** ^1^State Key Laboratory of Kidney Diseases, Department of Nephrology, National Clinical Research Center for Kidney Diseases, Chinese PLA General Hospital, Chinese PLA Institute of Nephrology, Beijing, China; ^2^Department of Ophthalmology, Ophthalmology and Visual Science Key Lab of PLA, Chinese PLA General Hospital, Beijing, China

**Keywords:** inflammation, mesangial proliferative glomerulonephritis, SOCS1, STAT1, MHC class II

## Abstract

Mesangial proliferative glomerulonephritis (MsGN) is a significant global threat to public health. Inflammation plays a crucial role in MsGN; however, the underlying mechanism remains unknown. Herein, we demonstrate that suppression of the cytokine signaling-1 (SOCS1)/signal transducer and activator of transcription 1 (STAT1) signaling pathway is associated with renal inflammation and renal injury in MsGN. Using MsGN rat (Thy1.1 GN) and mouse (Habu GN) models, renal SOCS1/STAT1 was determined to be associated with CD4+ T cell infiltration and related cytokines. *In vitro*, SOCS1 overexpression repressed IFN-γ-induced MHC class II and cytokine levels and STAT1 phosphorylation in mesangial cells. SOCS1 and STAT1 inhibitors significantly inhibited IFN-γ-induced CIITA promoter activity and MHC class II expression. In conclusion, our study emphasizes the pivotal role of the SOCS1/STAT1 axis in the regulation of inflammation in MsGN.

## Introduction

Glomerulonephritis (GN) is characterized by intraglomerular inflammation and is a major cause of end-stage renal disease (ESRD). Many forms of GN, such as immunoglobulin A nephropathy (IgAN), lupus nephritis (LN), and postinfectious endocapillary proliferative GN, are characterized by mesangial hyperplasia as a result of increased glomerular mesangial cell replication. Mesangial cell proliferation is a significant event in the development of progressive glomerular injuries; however, the common mechanisms underlying both of these important pathological findings remain unresolved.

Inflammation plays a crucial role in the progression of mesangial proliferative GN (MsGN). Several clinical and experimental studies have suggested that the number of lymphocytes in biopsy specimens from proliferative and nonproliferative GN is increased (1–3). CD4+ T lymphocytes not only regulate the immune system but also activate resident renal cells via the production and release of various cytokines (4, 5). T cell infiltration into the kidney is correlated with decreased renal function, heavy proteinuria, and severe glomerular, interstitial, and arterial lesions in patients with IgAN (6). T helper (Th) cytokines change the phenotype of mesangial cells, inducing and/or enhancing the expression of major histocompatibility complex (MHC) class II and chemokines (7). MHC antigen expression plays a pivotal role in antigen presentation and cellular immune reaction initiation. In normal murine kidneys, the basal expression levels of MHC class II are very low or absent (8). However, renal injury is associated with increased MHC molecule expression (9). The aberrant expression of MHC molecules, which activate infiltrating T cells, results in the initiation and promotion of inflammatory processes in glomeruli.

Suppressors of cytokine signaling (CIS and SOCS1–SOCS7) proteins are induced by cytokines and thereby act in a classical negative feedback loop to inhibit cytokine signal transduction. SOCS proteins are key physiological regulators of both innate and adaptive immunity (10). The SOCS1 protein is a key negative regulator of the Janus kinase (JAK)/STAT signaling pathway and has emerged as an interesting therapeutic target with anti-inflammatory actions (11). SOCS1 may also act as a key negative regulator of IL-4-induced IRS-2 signaling and M2 differentiation in human asthma (12, 13). Recent findings indicate that SOCS1 plays an important protective role, counteracting renal injury (14, 15).

SOCS proteins suppress proinflammatory factors and ameliorate renal injury in diabetic nephropathy (DN), LN and experimental immune-mediated nephritis (15–18). We hypothesized that SOCS1 may regulate inflammatory reactions in MsGN via the JAK-STAT pathway. Therefore, this study was performed to elucidate the role of SOCS1/STAT in regulating renal inflammation in MsGN models.

## Materials and methods

### Cell culture and transfection

Mouse mesangial cells (MMCs) were obtained from the American Type Culture Collection (Manassas, VA, USA). MMCs were cultured in DMEM/F-12 (Corning, NY, USA) supplemented with 5% FBS (HyClone, South Logan, UT, USA). Cells were made quiescent by culturing in serum-free medium for 24 h. In each experiment, MMCs were stimulated with 100 U/ml recombinant mouse IFN-γ (Sigma–Aldrich, St. Louis, MO, USA) (19). In several experiments, the STAT1 inhibitor fludarabine (25 μM; Sigma–Aldrich) was added to IFN-γ-treated MMCs (20). MMC transfection with SOCS1 plasmids (Generay, Shanghai, China) was performed using Lipofectamine 2000 according to the manufacturer's protocol (Invitrogen, Carlsbad, CA, USA). After 24 h of transfection, the cells were washed and stimulated with or without IFN-γ for 48 h.

### Animal experiments

Male C57BL/6 mice and Wistar rats were purchased from Peiking HuafuKang Biotech Co., Ltd. Habu GN was induced in 18- to 20-g C57BL/6 mice via intravenous (i.v.) tail injections of the snake toxin *Trimeresurus flavoviridis* (2.5 mg/kg body weight; Sigma–Aldrich). Moreover, 10 mice (per treatment group) were treated with phosphate-buffered saline (PBS) that contained the STAT1 inhibitor fludarabine (100 mg/kg, i.p.; Sigma–Aldrich) or vehicle (PBS) once every other day (21). Thy1.1 GN was induced in 180- to 200-g rats (*n* = 10 each) via a single i.v. tail vein injection of a Thy1.1 monoclonal antibody (mAb, 2.5 mg/kg), which was produced by OX-7 cells and diluted in 0.9% saline. The mice and rats were sacrificed immediately after the *Trimeresurus flavoviridis* or OX-7 mAb injection at 3, 5, 7, or 14 days after GN induction. At each time point, following euthanasia, renal tissue was obtained, and glomeruli were isolated by differential sieving (22, 23). The cortex was subsequently cut off with a fine pair of surgical scissors and placed in a plastic Petri dish that contained PBS buffer. The cortex was forced through sequential sieving (150, 106, and 75 μm for the rats; 125, 71, and 53 μm for the mice at room temperature (23, 24). The glomeruli were collected on the sieve (75 μm for the rats, 53 μm for the mice) using cold PBS buffer, centrifuged at 1,000 rpm for 5 min in a clinical centrifuge, resuspended in 5 ml of cold PBS buffer, and assessed for purity (more than 95% glomeruli, Figure S1) under the microscope. The glomeruli were maintained at −80°C until use for RNA and protein extraction. Kidneys were used for quantitative evaluation of glomerular damage [periodic acid-Schiff (PAS) staining] or immunohistochemistry. Blood urea nitrogen (BUN) and creatinine (Cr) levels were measured to assess renal function. This study was conducted in accordance with recommendations from the guidelines of the Hospital Research Ethics Committee, and written informed consent was obtained from all subjects in accordance with the Declaration of Helsinki. The protocol was approved by the Hospital Research Ethics Committee.

### Quantitative real-time PCR

Total RNA was extracted from MMCs and isolated glomeruli with TRIzol reagent (Invitrogen) according to the manufacturer's instructions. Total RNA was reverse-transcribed using a ReverTra Ace qPCR RT kit (Toyobo, Osaka, Japan). Quantitative real-time (qRT)-PCR was performed using SYBR Select Master Mix (Life Technologies, California, USA) and an RT-PCR detection system (ABI, Foster City, CA, USA). Primers were used to amplify the following genes (Supplementary Table 1): *MHC class II* molecules, *IFN-*γ*, TNF-*α*, IL-6, IL-12A, IL-12B, IL-17A, IL-23A (p19), IP-10, Mig, SOCS1, CIITA, and GAPDH*. Gene expression in the kidneys and cells was quantified with two-step RT-PCR, and the fold change in gene expression was calculated using the 2^−−ΔΔ^CT method. GAPDH was used as an internal loading control, and the target gene expression is presented as a ratio to GAPDH expression. All experiments were repeated at least three times.

### Western blotting

Proteins isolated from MMCs and isolated glomeruli samples were separated by SDS–PAGE and transferred to nitrocellulose (NC) membranes, blocked with 5% nonfat dried milk and incubated at 4°C overnight with primary antibodies, including MHC class II (Abcam, Cambridge, MA, USA), SOCS1 (Abcam), β-actin (Abcam), phosphorylated STAT1 (P-STAT1) and STAT1 antibodies (Cell Signaling Technology, Danvers, MA, USA). Secondary antibodies were subsequently applied, and the signals were detected using the ChemiDoc-It 600 Imaging System (UVP, Upland, USA). Densitometry analysis was performed using an ImageJ analysis system. All experiments were repeated at least three times.

### Flow cytometry

Serum-deprived, quiescent MMCs were treated with IFN-γ. The cells were detached, centrifuged at 500 g for 5 min and washed twice with stain buffer (FBS; BD Pharmingen, San Diego, CA, USA). The cell pellet was resuspended in 98 μl of stain buffer (FBS) and 2 μl of the fluorescence-conjugated mAb APC-anti-MHC-II (Miltenyi Biotec, Bergisch Gladbach, Germany). Isotype controls were used to confirm antibody specificity. The cells were incubated in the dark for 20 min at 4°C and then subsequently washed once in stain buffer (FBS), fixed in 400 μl of stain buffer (FBS) and analyzed for the presence of fluorescence-labeled donor cells using a Beckman flow cytometer (Miami, FL, USA).

### Histopathology and immunohistochemistry

Kidney sections (4 μm thick) were prepared using a routine procedure. Sections were stained with PAS reagent. To analyze the extent of mesangiolysis and the degree of mesangial matrix expansion and glomerular sclerosis, semiquantitative scoring systems (0–4) were used with the PAS-stained paraffin sections (100 systematically sampled glomeruli per animal, magnification × 400) as previously described (25, 26). Sections were used for immunohistochemical analysis with the following antibodies: proliferating cell nuclear antigen (PCNA; Cell Signaling Technology), anti-CD4 (Abcam), P-STAT1 (Cell Signaling Technology), and rabbit IgG polyclonal-Isotype Control (Abcam). The analysis was performed with the Vectastain Elite ABC-HRP kit according to the manufacturer's instructions (Vector Laboratories, USA). For immunofluorescence, the sections were incubated with SOCS1, CD4, CD68, and MHC class II, rabbit IgG polyclonal-Isotype Control, and rat IgG monoclonal-Isotype control (Abcam) antibodies followed by Alexa Fluor 555-conjugated secondary antibodies (Invitrogen). The sections were viewed with a microscope. The number of PCNA+, CD4+, CD68+, and P-STAT1+ cells per glomerular area was counted, and the positive staining was expressed as a percentage of the total area and number of positive cells [per glomerular cross-section (gcs) or per mm^2^].

### Luciferase reporter assays

The mouse CIITA IV promoter (−1404/+83 region) luciferase plasmids were generated according to a previous report (27). The MMCs were cotransfected with the CIITA IV reporter plasmid along with the pRL-TK Renilla plasmid (Promega) and 200 ng of SOCS1 expression plasmids or PCDNA3.1 plasmids. After promoter vector transfection, the cells were treated with IFN-γ or the STAT1 inhibitor fludarabine for 48 h. The luciferase assays were performed 48 h after transfection according to the dual-luciferase assay system protocol (Promega).

### Statistical analysis

Data were analyzed using SPSS 19.0 for Windows (SPSS Inc., Chicago, IL, USA) and are presented as the means ± SEMs. Statistical significance, defined as *P* < 0.05, was evaluated using Student's *t*-test or one-way ANOVA.

## Results

### Pathological features of rat and mouse MsGN models

**Figure 1:**
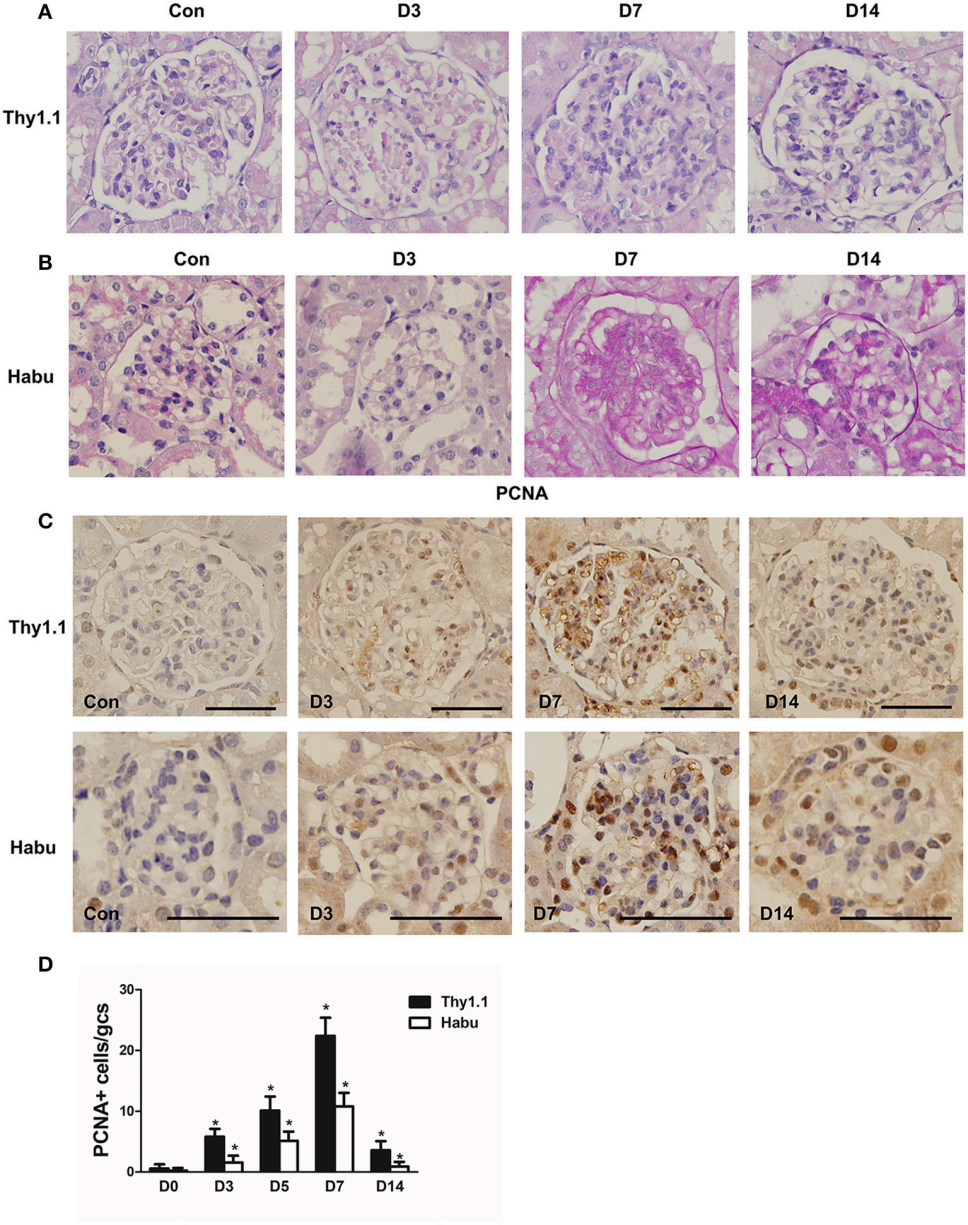
Pathological changes in glomeruli during each stage of rat Thy1.1 and murine Habu glomerulonephritis. **(A)** Periodic acid-Schiff (PAS)-stained tissues were examined during the time course of nephritis induced by anti-Thy l.1 antibody injections in rats. **(B)** PAS-stained tissues were examined during the time course of nephritis induced by Habu snake *Trimeresurus flavoviridis* injections in mice. **(C)** Glomerular cell proliferation, as shown by proliferating cell nuclear antigen (PCNA) staining, and histological analysis of PCNA-positive glomerular cells in kidney sections obtained from Thy1.1 and Habu GN models. **(D)** Quantification of PCNA-positive cells in glomeruli of MsGN models. Data are presented as the mean ± SEM (*n* = 10 per group). *P < 0.05, compared with the Con. Con: non-nephritic phosphate-buffered saline (PBS)-treated animals. Original magnification, × 400; Scale bar = 50 μm.

Thy1.1 GN, an antibody-induced, complement-mediated rat model that recapitulates the main features of human mesangial proliferative glomerular diseases, was generated. In addition, mouse GN was induced with Habu snake venom (HV). PAS staining was employed to assess the pathological changes throughout the course of Thy1.1 and Habu GN. Mesangiolysis and mesangial cell proliferation with mesangial expansion are the major pathological changes in these two MsGN models. The total glomerular cellularity was decreased during the acute mesangiolysis stage on day (D) 3 compared with that in the healthy animals. Mesangial cell proliferation with extracellular matrix (ECM) accumulation was apparent on D7; this mesangioproliferative lesion mostly resolved on D14 with several segmental increases in the mesangial matrix (Figures 1A,B). Cell proliferation in the glomeruli was determined by PCNA immunohistochemistry. The PCNA staining results indicated that the cell proliferation rate was substantially increased on D7 in the Thy1.1 and Habu GNs (Figures 1C,D). Taken together, these results indicate that the Thy1.1 and Habu GN models were successfully established.

### Glomerular infiltrating inflammatory cells and cytokines in the MsGN model

**Figure 2:**
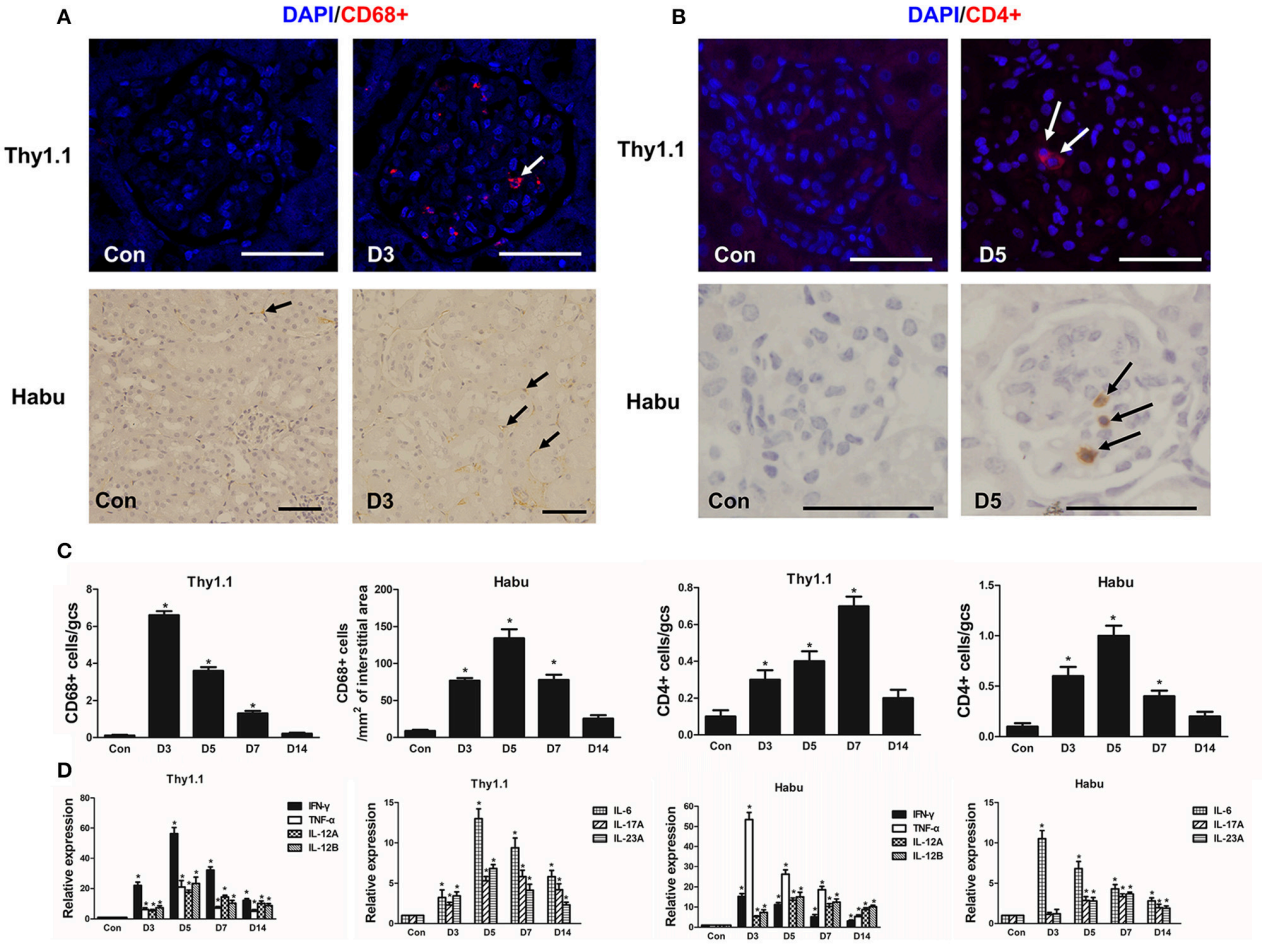
Time course of inflammatory cell and cytokine glomerular infiltration after MsGN model establishment. **(A)** Histological analysis of CD68+ macrophages (arrows) in glomeruli and interstitium from Thy1.1 GN and Habu GN. **(B)** Histological analysis of CD4+ T cells (arrows) in glomeruli from Thy1.1 and Habu GN. **(C)** Quantification of CD68+ macrophages and CD4+ T cells in Thy1.1 GN and Habu GN. **(D)** mRNA expression of *IFN-*γ, *TNF-*α, *IL-12A, IL-12B, IL-6, IL-17A, and IL-23A* in glomeruli was detected via RT-PCR; PCR data are normalized to *GAPDH*. Data are presented as the mean ± SEM (*n* = 10 per group). The results are representative of three independent experiments. *P < 0.05 versus Con. Con: non-nephritic phosphate-buffered saline (PBS)-treated animals. Original magnification, × 400; Scale bar = 50 μm.

The number of CD68+ macrophages per gcs was significantly increased in glomeruli and peaked on D3 in Thy1.1 GN (Figures 2A,C). In contrast, in Habu GN, CD68+ macrophage infiltration was mainly identified in the renal interstitial space instead of the glomeruli (Figures 2A,C). In both MsGN models, the number of CD4+ T lymphocytes was significantly increased, with a peak that appeared on D7 in Thy1.1 GN and on D5 in Habu GN (Figures 2B,C). IFN-γ, TNF-α, IL-12, IL-6, IL-17, and IL-23 are mainly produced by T cells and macrophages and play roles in renal damage progression. Thus, we analyzed the mRNA expression of these cytokines in the kidney. IL-12 is a heterodimeric cytokine encoded by two separate genes: *IL-12A* (p35) and *IL-12B* (p40). *IFN-*γ*, TNF-*α*, IL-12A, IL-12B, IL-6, IL-17A, and IL-23A* expression was significantly increased throughout the course of Thy1.1 and Habu GN and significantly decreased on D14 (Figure 2D). These data indicate that inflammatory cells and cytokines participate in MsGN.

### Glomerular expression of MHC class II in MsGN models

**Figure 3:**
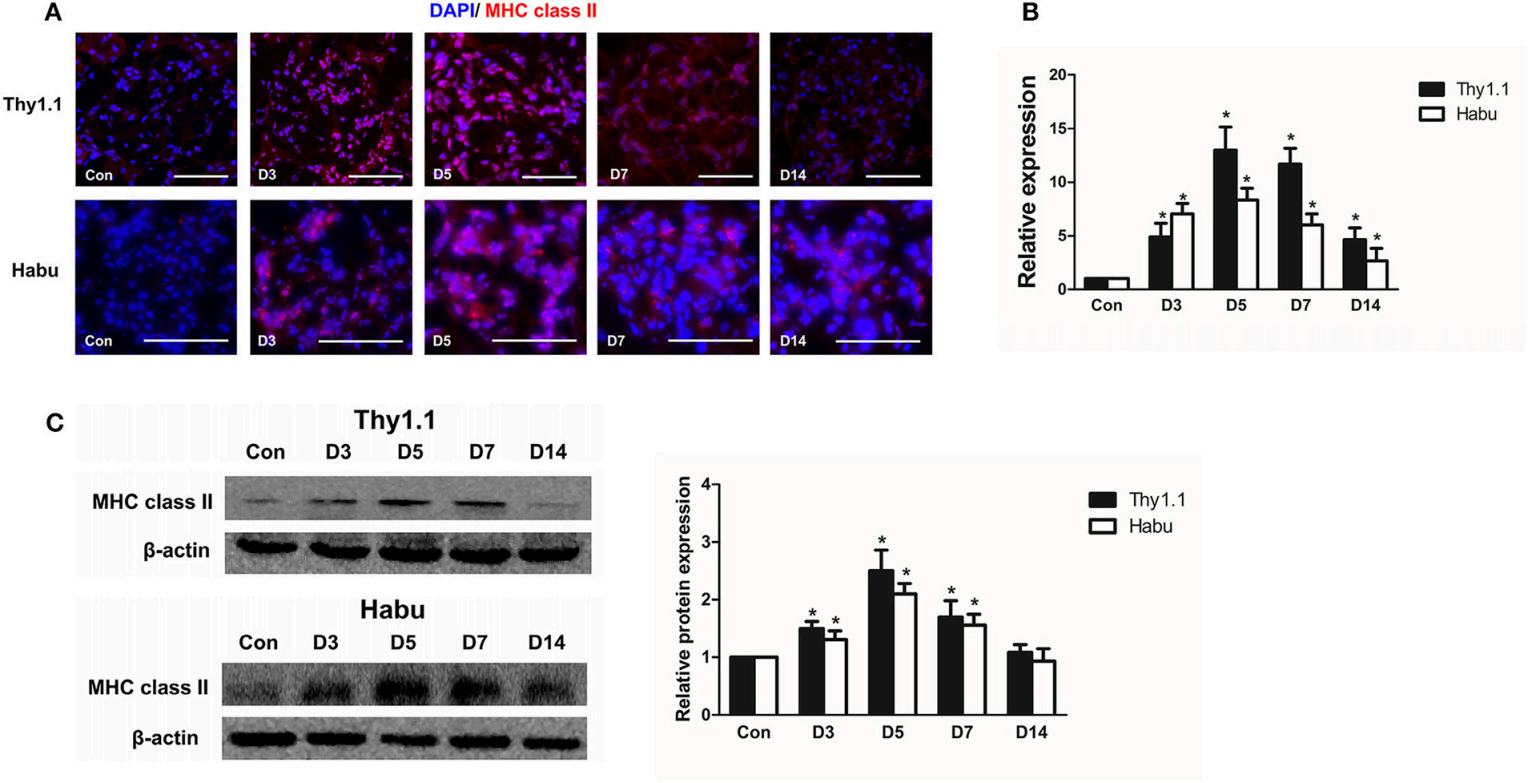
Glomerular expression of MHC class II in MsGN models. **(A)** Immunofluorescence analysis was performed on 4-μm-thick kidney sections to determine MHC class II expression in glomeruli. **(B)** Quantitative RT-PCR analysis of *MHC class II* mRNA in glomeruli from MsGN models. **(C)** Representative Western blots showing the levels of MHC class II protein expression in glomeruli of MsGN models. Data are presented as the mean ± SEM (*n* = 10 per group). The results are representative of three independent experiments. *P < 0.05 versus Con. Con: non-nephritic phosphate-buffered saline (PBS)-treated animals. Original magnification, × 400; Scale bar = 50 μm.

Previous findings have indicated that the upregulation of MHC class II molecules on mesangial cells is associated with renal dysfunction in the pathogenesis of acute murine malarial nephritis (28). We investigated the expression of MHC class II in MsGN models. Immunofluorescence staining showed that MHC class II was strongly expressed in glomeruli throughout the course of the MsGN models (Figure 3A); however, it was absent in the kidneys of the normal groups. In RNA isolated from glomeruli, the MHC class II mRNA levels were upregulated in the animals with MsGN compared with those in the normal animals (Figure 3B). The MHC class II protein expression was conspicuously increased in the MsGN kidneys compared with that in the normal kidneys (Figure 3C). Mesangial cells may have the potential to function as antigen-presenting cells (APCs) and activate infiltrating T cells to propagate a series of cellular immune reactions in MsGN.

### SOCS1 and STAT1 in the kidneys of MsGN models

**Figure 4:**
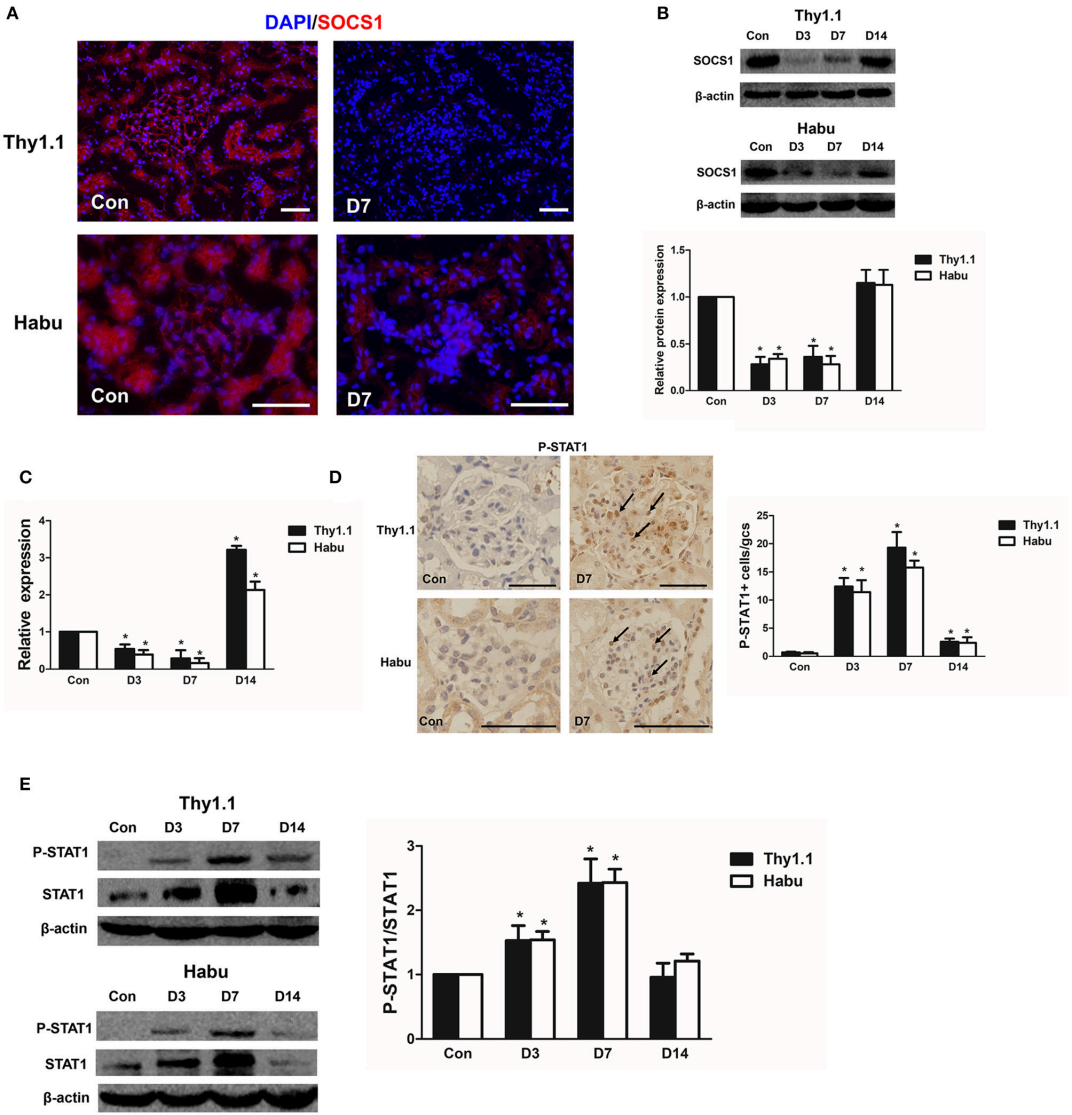
SOCS1 and STAT1 expression in MsGN models. **(A)** Immunofluorescence analysis was performed to determine SOCS1 expression in Thy1.1 and Habu GN glomeruli (Thy1.1: magnification × 200; Habu: magnification × 400). **(B)** SOCS1 protein expression in Thy1.1 and Habu GN glomeruli. Quantitative analysis of SOCS1 protein expression in glomeruli of MsGN models. **(C)** Quantitative RT-PCR analysis of *SOCS1* mRNA expression in glomeruli of MsGN models. **(D)** Immunohistochemistry analysis of P-STAT1 in Thy1.1 and Habu GN kidney sections. Representative micrographs and quantification of positive cells in glomeruli are shown (original magnification, × 400). **(E)** Western blot analysis of P-STAT1 and STAT1 expression in Thy1.1 and Habu GN glomeruli; representative images and the summary of the normalized quantification are shown. Data are presented as the mean ± SEM (*n* = 10 per group). The results are representative of three independent experiments. *P < 0.05 vs. Con. Con: non-nephritic phosphate-buffered saline (PBS)-treated animals. Scale bar = 50 μm.

SOCS1/STAT1 contribute to the regulation of renal inflammation in DN (16). Thus, we analyzed the time course of SOCS1 and STAT1 expression during the progression of the MsGN models. SOCS1 immunofluorescence staining was decreased in the kidneys of the MsGN models compared with that in the healthy animals (Figure 4A, Figure S2). The SOCS1 protein and mRNA expression was also significantly decreased during the early stages of the MsGN models (Figures 4B,C, Figure S4). Furthermore, immunohistochemical analysis to detect STAT1 protein activation indicated intense nuclear staining of phosphorylated STAT1 (P-STAT1) in glomeruli during MsGN model progression (Figure 4D, Figure S3). Western blot analysis showed that STAT1 and P-STAT1 expression were significantly increased in the MsGN models (Figure 4E). These data suggest that SOCS1/STAT1 participate in MsGN model progression.

### STAT1 inhibitor decreases renal inflammation and ameliorates glomerular lesions in Habu GN

**Figure 5:**
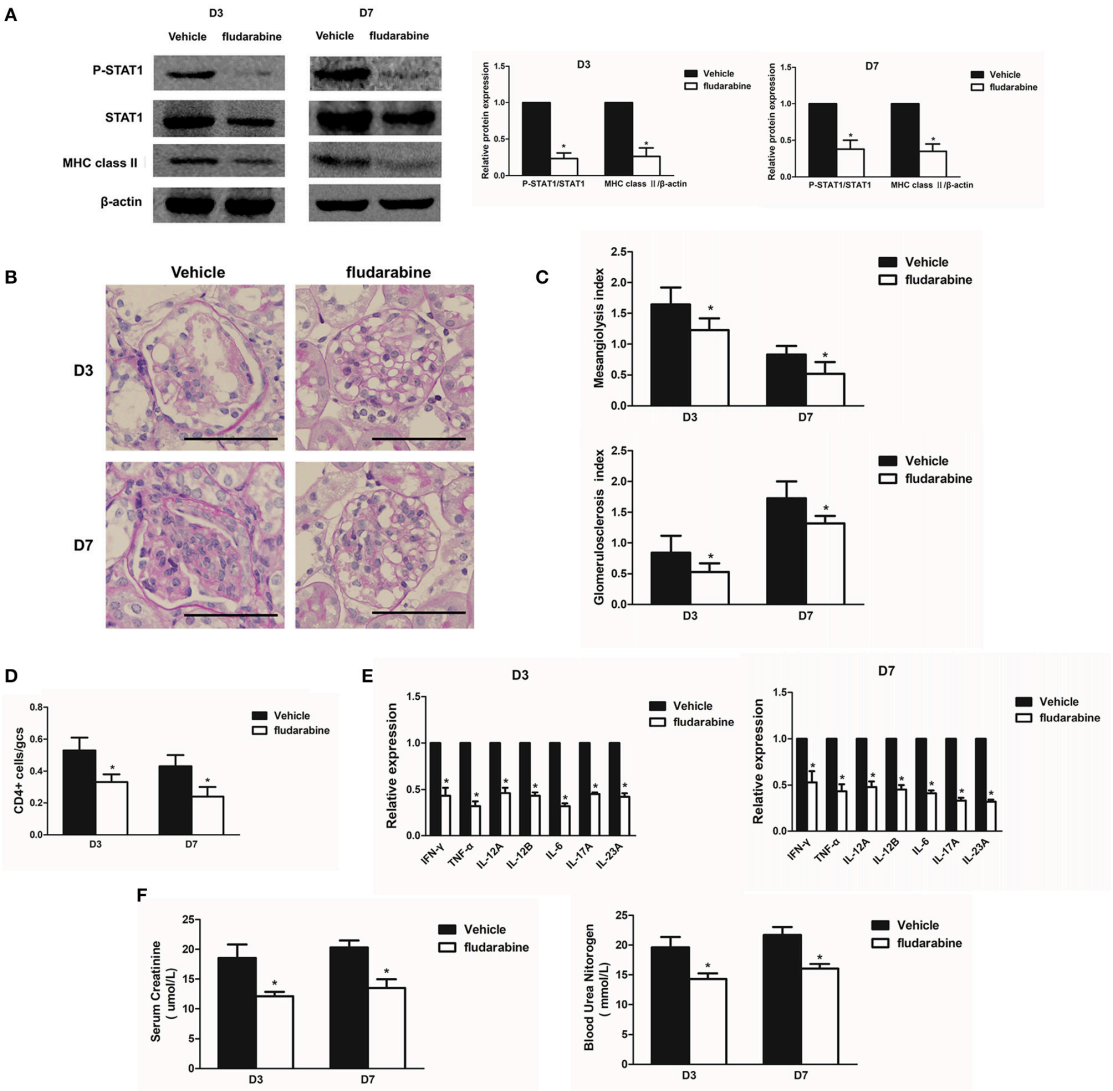
Treatment with the STAT1 inhibitor fludarabine reduces renal inflammation and ameliorates glomerular lesions in Habu GN. **(A)** Western blots showing P-STAT1, STAT1, and MHC class II expression levels in glomeruli of Habu GN, which were treated with the STAT1 inhibitor fludarabine, on days 3 and 7; representative images and a summary of the normalized quantification are shown. **(B)** PAS-stained kidney tissues were examined in Habu GN treated with fludarabine on days 3 and 7. **(C)** Mesangiolysis index and glomerulosclerosis index in the fludarabine-treated Habu GN group compared with those in the control group on days 3 and 7. **(D)** CD4+ T cell infiltration was suppressed in the fludarabine-treated group compared with that in the control group on days 3 and 7. **(E)** Relative *IFN-*γ, *TNF-*α, *IL-12A, IL-12B, IL-6, IL-17A. and IL-23A* mRNA levels in the fludarabine-treated Habu GN glomeruli as determined by RT-PCR. Data are presented as the mean ± SEM. **(F)** Effects of fludarabine on plasma creatinine and blood urea nitrogen (BUN) levels measured in Habu GN on D3 and D7. Data are presented as the mean ± SEM (*n* = 10 per group). The results are representative of three independent experiments. *P < 0.05 versus vehicle. Vehicle: PBS-treated Habu GN. Original magnification, × 400; Scale bar = 50 μm.

To explore the role of STAT1 in renal inflammation and MsGN development, the Habu GN groups were treated with fludarabine, a pharmacological inhibitor that has been reported to specifically inhibit STAT1 (21). The results indicated that fludarabine treatment significantly prevented STAT1 phosphorylation and inhibited MHC class II expression in the Habu GN group, as determined by Western blot analysis (Figure 5A). The increased mesangiolysis index and mesangial matrix expansion in the Habu GN model was significantly lower in the fludarabine-treated groups than in the untreated groups on D3 and D7 (Figures 5B, C). The fludarabine-treated mice presented significantly reduced numbers of infiltrating CD4+ T lymphocytes (Figure 5D). Furthermore, fludarabine treatment decreased the gene expression levels of *IFN-*γ*, TNF-*α, *IL-12, IL-6, IL-17A*, and *IL-23A* in the glomeruli of the Habu GN model mice (Figure 5E). The BUN and Cr levels in the fludarabine-treated mice were significantly lower than those in vehicle group mice (Figure 5F). These results demonstrate that the STAT1 inhibitor decreases renal inflammation and ameliorates glomerular lesions in Habu GN.

### SOCS1 inhibits MHC class II and chemokine expression in mesangial cells

**Figure 6:**
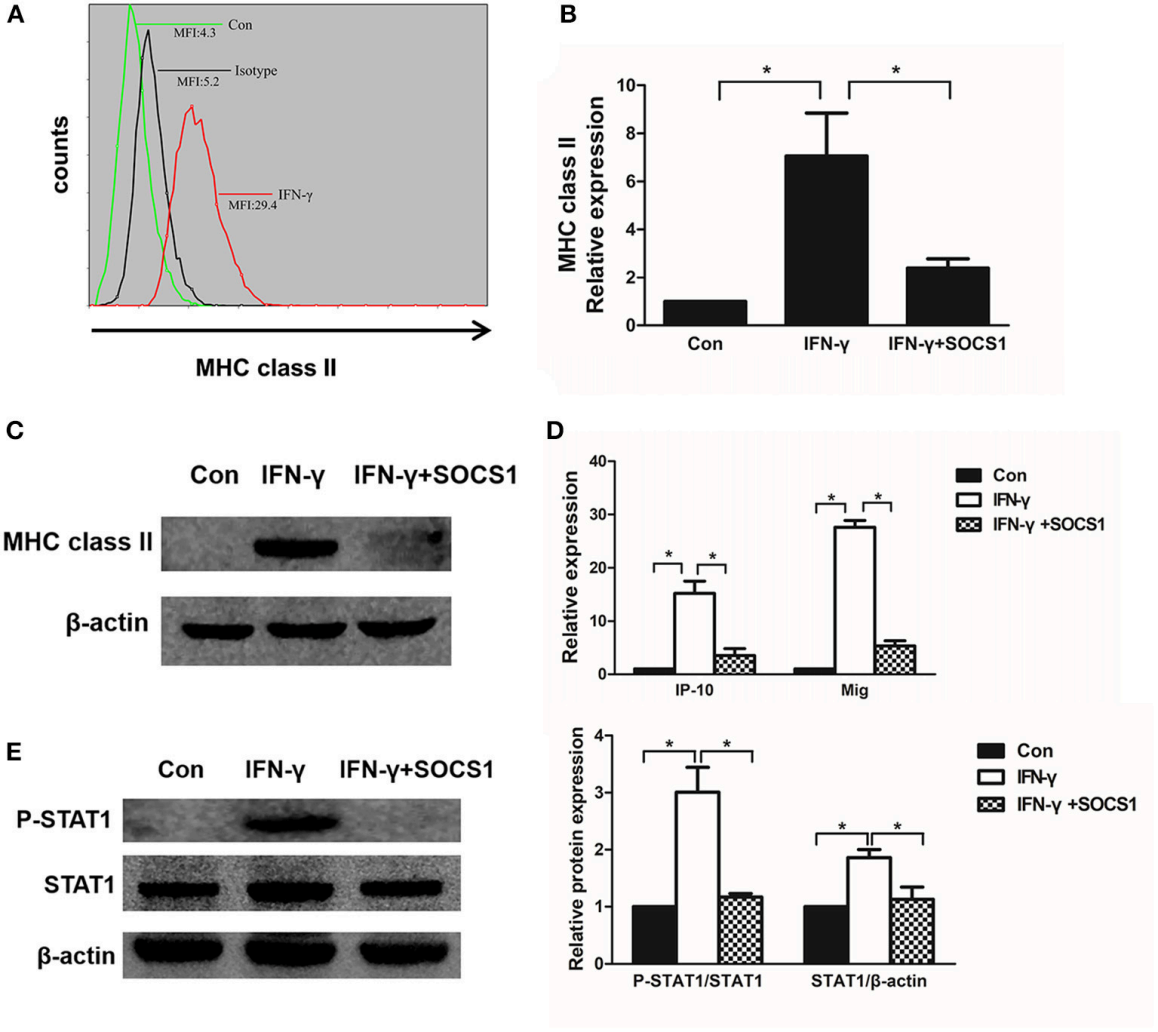
SOCS1 expression inhibits IFN-γ-induced MHC class II expression and chemokine production. **(A)** Flow cytometric analysis of MHC class II expression in IFN-γ-stimulated mesangial cells. **(B)** Quantitative RT-PCR analysis of *MHC class II* mRNA expression in IFN-γ-stimulated MMCs transfected with SOCS1 plasmids. **(C)** MMCs transfected with SOCS1 plasmids were incubated in the absence or presence of IFN-γ for 48 h. Representative Western blots show MHC class II levels in IFN-γ-stimulated mesangial cells. **(D)** Relative *IP-10* and *Mig* mRNA levels in IFN-γ-treated MMCs transfected with SOCS1 plasmids as determined by RT-PCR. **(E)** Western blot analysis of P-STAT1 and STAT1 expression in SOCS1 plasmid-transfected MMCs incubated in the absence or presence of IFN-γ for 48 h; representative images and the summary of the normalized quantification are shown. Data are presented as the mean ± SEM (*n* = 3). The results are representative of three independent experiments. *P < 0.05 vs. Con.

MHC class II molecules present exogenous antigenic peptides to CD4+ T lymphocytes. IFN-γ induced chemokine production and the cell surface expression of MHC class II molecules in mesangial cells (29, 30). After stimulation with IFN-γ for 48 h, we determined that the MHC class II expression was increased on the surface of MMCs via flow cytometry (Figure 6A). To investigate the effects of SOCS1 on MHC class II expression in IFN-γ-stimulated MMCs, we transfected SOCS1 overexpression plasmids into MMCs and determined that ectopic SOCS1 expression significantly decreased the *MHC class II* mRNA and protein levels (Figures 6B, C). We subsequently assessed whether SOCS1 can suppress related chemokine expression in MMCs. IP-10 and Mig are two chemokines expressed by IFN-γ-treated mesangial cells under inflammatory conditions that are activated by the JAK/STAT1 signaling pathway (31). SOCS1 overexpression substantially repressed *IP-10* and *Mig* expression in the IFN-γ-stimulated MMCs (Figure 6D). We also showed that SOCS1 overexpression repressed IFN-γ-induced STAT1 phosphorylation in the MMCs (Figure 6E). These *in vitro* data verified the significant inhibitory role of SOCS1 on MHC class II expression and chemokine expression in mesangial cells.

### Effect of STAT1 inhibitor on MHC class II and chemokine expression in mesangial cells

**Figure 7:**
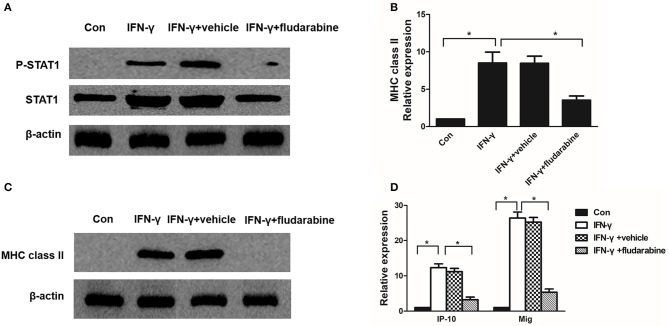
Fludarabine inhibits IFN-γ-induced MHC class II expression and chemokine production. **(A)** Western blot analysis of P-STAT1 and STAT1 in IFN-γ-treated MMCs in the absence or presence of fludarabine for 48 h. **(B)** Quantitative RT-PCR analysis of *MHC class II* mRNA expression in IFN-γ-stimulated MMCs in the absence or presence of fludarabine for 48 h. **(C)** Representative Western blots showing the levels of MHC class II in IFN-γ-stimulated mesangial cells in the absence or presence of fludarabine for 48 h. **(D)** Relative levels of *IP-10* and *Mig* mRNA in IFN-γ-treated MMCs in the absence or presence of fludarabine for 48 h as determined by RT-PCR. Data are presented as the mean ± SEM (*n* = 3). The results are representative of three independent experiments. *P < 0.05 vs. Con. Vehicle: PBS.

To determine whether STAT1 regulates MHC class II and chemokine expression in mesangial cells, we used the STAT1 inhibitor fludarabine in IFN-γ-treated mesangial cells. Fludarabine prevented IFN-γ-induced STAT1 phosphorylation (Figure 7A). Fludarabine substantially repressed *MHC class II* mRNA and protein expression in the IFN-γ-treated MMC cells (Figures 7B, C). In addition, fludarabine inhibited IFN-γ-induced *IP-10* and *Mig* expression in the MMCs (Figure 7D). In summary, the STAT1 inhibitor repressed MHC class II and chemokine expression, which correlated with decreased P-STAT1 levels.

### SOCS1 inhibits IFN-γ-induced CIITA promoter IV activity and gene expression by inhibiting STAT1

**Figure 8:**
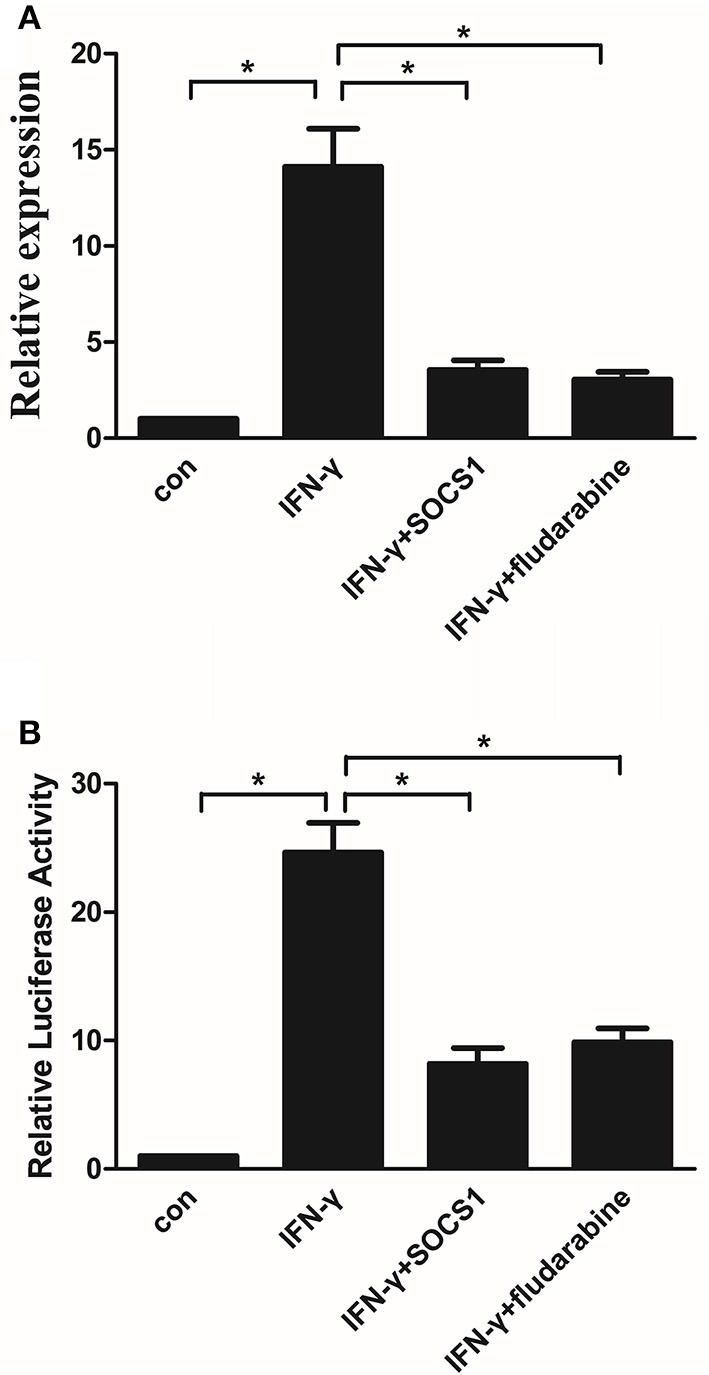
SOCS-1 inhibits IFN-γ-induced CIITA promoter IV activity and gene expression via STAT1 regulation. **(A)** Quantitative RT-PCR analysis of *CIITA* mRNA expression in MMCs transfected with SOCS1 plasmids or treated with fludarabine for 48 h incubated in the absence or presence of IFN-γ for 48 h. **(B)** MMCs were cotransfected with the CIITA promoter and SOCS1 plasmid or treated with fludarabine for 48 h in the absence or presence of IFN-γ for 48 h prior to analyze for luciferase activity as indicated in the Materials and Methods. Statistical significance was determined relative to the negative control. Data are presented as the mean ± SEM (*n* = 3) of at least three experiments. *P < 0.05 vs. Con.

CIITA, which is inducible by IFN-γ, acts as a general regulator of *MHC-II* gene expression (27). We investigated the effects of the SOCS1 protein and STAT1 inhibitor on IFN-γ-induced CIITA promoter IV activity and gene expression in MMCs. IFN-γ treatment increased the *CIITA* mRNA expression in the mock-transfected cells, whereas SOCS-1 overexpression significantly inhibited IFN-γ-induced CIITA expression (Figure 8A). IFN-γ treatment increased the CIITA promoter IV activity in the mock-transfected cells, and SOCS-1 overexpression decreased the IFN-γ-induced CIITA promoter IV activity (Figure 8B). The STAT1 inhibitor significantly inhibited IFN-γ-induced *CIITA* expression and promoter activity (Figures 8A, B). In summary, SOCS-1 expression inhibited IFN-γ-induced CIITA promoter IV activation by inhibiting the tyrosine phosphorylation of STAT1.

## Discussion

This study demonstrates that SOCS1 expression is decreased in the kidneys of MsGN model animals and that kidney inflammatory cell recruitment, proinflammatory factor expression levels, and STAT1 activation are increased. Moreover, MHC class II was strongly expressed in glomeruli of the MsGN models. In mesangial cells, SOCS1 expression inhibited STAT1 protein expression, and IFN-γ induced STAT1 phosphorylation and MHC class II expression.

To explore the role of SOCS1 in regulating proinflammatory factors and STAT1 activation in MsGN, we established rat Thy1.1 and mouse Habu GN models. Thy1.1 GN is induced by passively injecting an anti-Thy1.1 mAb, which binds to target antigens on the mesangial cell surface and causes mesangiolysis within 24 to 48 h. After mesangiolysis, the infiltration of inflammatory cells, such as lymphocytes and macrophages, and consequential mesangial cell proliferation were identified (32). As MMCs lack the Thy1.1 antigen, the Thy1.1 GN model cannot be induced in mice. Thus, the widely used, well-established model of MsGN in mice is Habu GN (33). The venom destroys the mesangial structure. However, the damage typically heals spontaneously, as mesangial cells actively participate in regeneration, and this process is accompanied by inflammatory cell infiltration in the early course of Habu GN (26). The Thy1.1 and Habu models are both MsGN models, and IgA nephropathy is the most common type of MsGN in humans. Although the IgA levels are not increased in these two models, they are widely used models of experimental MsGN. Understanding these processes of mesangial cell injury and recovery may provide insights into the pathogenesis of human mesangial cell diseases, such as IgA nephropathy.

T cells are directly involved in the renal pathology of MsGN. One study indicated that T cell reactivity with antigens in the mesangium is sufficient to initiate glomerular pathology (34). A retrospective study suggested that severe interstitial CD3+ cell infiltration indicated poor IgAN outcomes (35). An increase in the number of lymphocytes in the mesangial area seems to be involved in mesangial cell proliferation and the consequential alterations to the mesangium in Thy1.1 GN. Anti-CD5 mAb treatment suppressed CD4+ T cell recruitment into glomeruli and reduced proteinuria and mesangial injury (36). KAWACHI showed that continuous treatment with anti-CD4 reduced proteinuria and ameliorated the glomerular lesions in the experimental progressive GN. Depletion of CD4+ T cells also reduced the glomerular accumulation of CD8+ T cells and ED3+-activated macrophages and reduced the glomerular expression of IFN-γ mRNA (37). CD4+ T cell infiltration in the glomeruli was accompanied by upregulation of MHC class II expression in acute proliferative GN (38). Our results indicated that fludarabine inhibited MHC class II expression in Habu GN, decreased the number of infiltrating CD4+ T lymphocytes and ameliorated glomerular lesions. These studies indicated that MHC class II upregulation mainly contributes to the pathogenesis of MsGN models via the activation of CD4+ T cells.

In our models, circulating lymphocytes are recruited into glomeruli. The levels of inflammatory cytokines, such as *IFN-*γ, *TNF-*α, and *IL-12*, which are thought to be produced by T cells and macrophages, were significantly increased. These findings also support the idea that inflammatory cells are recruited into glomeruli. *IFN-*γ mRNA expression was substantially increased in the IgAN group compared with that in the non-IgAN group (39). TNF-α is an important mediator of IgAN pathogenesis (40) that is produced early in the inflammatory process and leads to the release of proinflammatory factors, including IL-6 and TNF-α (41). IFN-γ is the most highly upregulated cytokine in the Thy1.1 model, whereas TNF-α is the most highly upregulated cytokine in the Habu model. These two models appear to be differentially regulated. IFN-γ is considered to be produced by lymphocytes, and this finding supports the idea that lymphocytes are recruited into glomeruli. IFN-γ reportedly acts as an activating factor for macrophages (42), which may be the reason why CD68+ macrophages accumulated in glomeruli in Thy1.1 GN. However, in Habu GN, CD68+ macrophage infiltration was mainly identified in the renal interstitial space instead of glomeruli. TNF-α is produced from mononuclear cells, and it induces many biological activities such as inducing angiogenesis that are crucial for the development of GN (43). In the acute phase of the Habu model, impairment of mesangial integrity and the loss of endothelial cells lead to ballooning of glomerular capillaries, which are induced by snake venom. TNF-α expression in mesangiolytic glomeruli may induce the angiogenesis of Habu models, thus favoring repair or healing of the lesion. IL-12 administration significantly increases crescent formation, with glomerular macrophage accumulation and interstitial cell infiltration, in a high IgA inbred strain of ddY mice (44). Inflammatory cells and cytokines may play an important role in MsGN development.

IFN-γ, a pleiotropic cytokine produced principally by CD4+ cells, CD8+ T cells, and natural killer (NK) cells, is produced predominately by CD8 cytotoxic T cells, as determined by flow cytometry and ELISA (45). However, our study did not detect the infiltration of CD8+ and NK cells in MsGN models, and the numbers of infiltrating CD8+ T and NK cells were greater than those of CD4+ T cells in a Thy1.1 model reported in a previous study (36). Thus, we speculated that CD8+ T cells are the main producer of IFN-γ. However, fewer CD4+ T cells than CD8+ cells were found to infiltrate glomeruli, clearly showing that the initial recruitment of CD4+ T cells controlled the consequential recruitment and/or activation of CD8+ T cells and macrophages, which also contributed to the progression of irreversible GN (37). Taken together, these results suggest that CD4+ T cells, CD8+ T cells, and NK cells play an important role in producing IFN-γ in MsGN.

Mesangial cells have been identified as potential nonprofessional APCs. Mesangial cells express MHC class II in a rat crescentic GN model (7). Mesangial cells expressed readily detectable amounts of MHC class I on their surfaces, and IFN-γ strongly enhanced its expression (46, 47). MHC class I molecules can present antigens to CD8+ cytotoxic T cells. These reports indicated that CD8+ T cell infiltration significantly increased during the development of Thy1.1 GN (36, 48). Crosspresentation capacity has been shown for macrophages, dendritic cells (DCs) and nonhematopoietic cell types. Podocytes are considered to be nonhematopoietic professional APCs that activate CD4+ T cells and crosspresent exogenous antigens on MHC class I molecules to CD8+ T cells. Like DCs and podocytes, mesangial cells may also have the crosspresentation ability (49), which might explain why CD8+ T cell infiltration produced IFN-γ in the glomeruli of MsGN models.

SOCS proteins, which are inhibitors of the cytokine signaling pathways, unveiled an important mechanism for the negative regulation of the cytokine-induced JAK/STAT pathway. The protein expression level of SOCS1 was reported to be low in the absence of inflammatory stimulation, and upregulation of SOCS1 expression was observed after STAT1 phosphorylation (50). Our study showed that SOCS1 exhibited a high level of expression in normal rat and mouse kidneys. Several studies have also reported that SOCS1 is highly expressed in normal kidneys. Illei's study showed that SOCS1 expression was decreased in human kidney interstitial fibrosis of LN, and they indicated that miR-150 was upregulated in mesangial cells by currently unidentified factors. miR-150 directly decreases SOCS1 levels, which leads to the upregulation of profibrotic protein production in all three types of renal cells of LN (15). Xia's study indicated that the SOCS1 protein is expressed at high levels in the kidneys of normal human and mouse controls but significantly decreased in patients with LN or Mrl/lpr mice (51). We showed that SOCS1 expression was decreased on 3 and 7 days of the MsGN models. These two MsGN models are acute self-resolving disease models; we hypothesize that SOCS1 expression may increase renal damage in the two MsGN models at a stage earlier than 3 days by TLR signaling. The expression of SOCS1 decreased during the progression of the disease, whereas the expression level of SOCS1 was increased after STAT1 phosphorylation was activated.

SOCS1 participates in renal fibrosis by downregulating JAK2/STAT1-mediated cytokine signaling in LN (51). STAT1 is overexpressed in glomerular mesangial, endothelial, and tubular epithelial cells, whereas SOCS1 is downregulated accordingly in MRL/lpr mice (52). It has been suggested that the dysregulation of SOCS1 might be associated with the pathogenesis of SLE in a Chinese population (53). SOCS1 downregulation significantly increased the expression of profibrotic proteins in MMCs (15). An SOCS1 peptidomimetic has been used to halt the onset and progression of renal inflammation and fibrosis in DN (16). Consistent with previous kidney studies in LN and DN models, our results in two well-established MsGN models demonstrate that decreased SOCS1 expression and STAT1 activation are associated with renal inflammation and renal injury, thus confirming the key role of the SOCS1/STAT1 axis in MsGN pathogenesis.

STAT1 is preferentially active in DN and IgAN (54, 55) and plays a critical role in the signal transduction of IFN-γ in mesangial cells (56). Our results demonstrate that the STAT1 inhibitor fludarabine reduced renal inflammation and ameliorated renal injury in MsGN. When a murine model of SLE was treated with fludarabine, the renal pathology was significantly reduced in the treated mice compared with that in the control mice (57). A brief course of low-dose fludarabine and cyclophosphamide induces long-lasting remission in patients with proliferative LN (58). Moreover, a previous study demonstrated that fludarabine treatment attenuates AngII-induced leukocyte and macrophage infiltration and inflammatory cytokine expression (21). Our results suggest that fludarabine significantly decreased the expression of cytokines, including IFN-γ, TNF-α, and IL-12, which are produced by T cells and macrophages in the kidney. Thus, we speculated that the STAT1 inhibitor may selectively affect inflammatory cells and alleviate local renal injury in the kidney.

IFN-γ-upregulated CIITA and MHC class II expression via the STAT1 pathway is a critical process in antigen presentation and in the consequential activation of T cell-mediated immune reactions (59). Our findings indicate that SOCS1 and fludarabine inhibit IFN-γ-induced CIITA promoter activity and MHC class II expression by inhibiting IFN-γ-induced tyrosine STAT1 phosphorylation in mesangial cells. CIITA is a critical transcription factor for MHC class II expression, and CIITA expression is induced by IFN-γ in mesangial cells (60). STAT1 activation is the most essential, critical action in the signaling process for generating a cellular response to IFN-γ (59). IP-10 and Mig are induced in mesangial cells by the Th1 cytokine IFN-γ, and the glomerular attraction of T cells by locally produced IP-10 and Mig might play an important pathophysiological role in this process (61). Our data indicate that SOCS1 inhibits the IFN-γ-induced expression of *IP-10* and *Mig* in mesangial cells by targeting the JAK/STAT signaling pathway. Our results also indicate that SOCS1 may be responsible for attenuating IFN-γ-induced CIITA, MHC class II and chemokine expression via STAT1 inhibition in mesangial cells. The limitation of this study, resulting from a lack of specific markers in mesangial cells, is that whether mesangial cells secrete cytokines and are activated via the STAT1 signaling pathway in the animal model is not clear. We investigated these hypotheses using only cell experiments *in vitro*.

In conclusion, our study emphasizes the pivotal role of the SOCS1/STAT1 axis in the regulation of MsGN inflammation. In addition, these results demonstrate that SOCS1 is a critical regulator of cellular sensitivity to IFN-γ-induced CIITA and MHC class II expression in mesangial cells. The negative feedback between SOCS1/STAT1 expression and inflammation may play an important role in MsGN.

## Ethics statement

This study was carried out in accordance with the recommendations of the guidelines of the Hospital Research Ethics Committee with written informed consent from all subjects. All subjects gave written informed consent in accordance with the Declaration of Helsinki. The protocol was approved by the Hospital Research Ethics Committee.

## Author contributions

XMC and GC designed the research. JB, LLW, XNC, LQW, QL, and JW performed the research and analyzed the data. YZ contributed new reagents and analytical tools. JB and XMC wrote the paper.

### Conflict of interest statement

The authors declare that the research was conducted in the absence of any commercial or financial relationships that could be construed as a potential conflict of interest.

## Supplementary material

Figure S1 Microscopy of isolated rat **(A)** and mouse **(B)** glomeruli. Magnification, × 100.

Figure S2 An IgG isotype control antibody was used on 4-μm-thick kidney sections to determine MHC class II expression in Thy1.1 and Habu GN glomeruli via immunofluorescence. Original magnification, × 400, Scale bar = 50 μm.

Figure S3 An IgG isotype control antibody was used on kidney sections to determine P-STAT1 in Thy1.1 expression in Habu GN glomeruli via immunohistochemistry. Original magnification, × 400, Scale bar = 50 μm.

Figure S4 SOCS1 protein expression was detected in glomeruli of MsGN models. **(A)** SOCS1 protein expression was detected in glomeruli of Habu models using an SOCS1 antibody from Santa Cruz Biotechnology. **(B)** SOCS1 protein expression was detected in glomeruli of Thy1.1 and Habu models using an SOCS1 antibody from Abcam.

Supplementary Table 1 List of primers used in reverse transcriptase–polymerase chain reaction.

## Abbreviations

MsGN: mesangial proliferative glomerulonephritis
SOCS1: suppressor of cytokine signaling-1
ESRD: end-stage renal disease
IgAN: immunoglobulin A nephropathy
LN: lupus nephritis
MHC: major histocompatibility complex
Th: T helper
JAK: Janus kinase
DN: diabetic nephropathy
MMCs: mouse mesangial cells
qRT-PCR: quantitative real-time PCR
MFI: mean fluorescence intensity
P-STAT1: phosphorylated STAT1
HV: Habu snake venom
APCs: antigen-presenting cells
ECM: extracellular matrix.
