# Supplementary figures and images for: Suppressor of Cytokine Signaling-1/STAT1 Regulates Renal Inflammation in Mesangial Proliferative Glomerulonephritis Models
Source: Front Immunol. 2018 Aug 30;9:1982. doi: 10.3389/fimmu.2018.01982 (PMC6125399; doi:10.3389/fimmu.2018.01982)

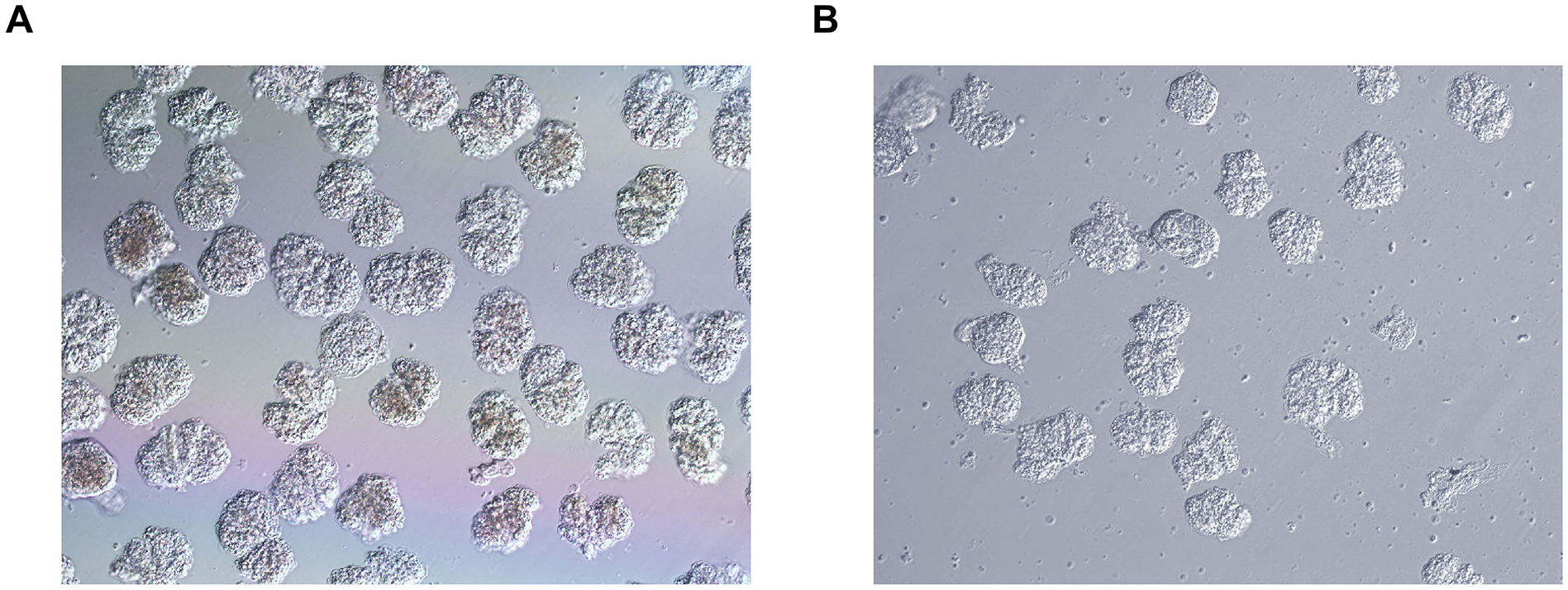

Supplement: Figure S1 — Microscopy of isolated rat (A) and mouse (B) glomeruli. Magnification, × 100. [file Image_1.TIF]

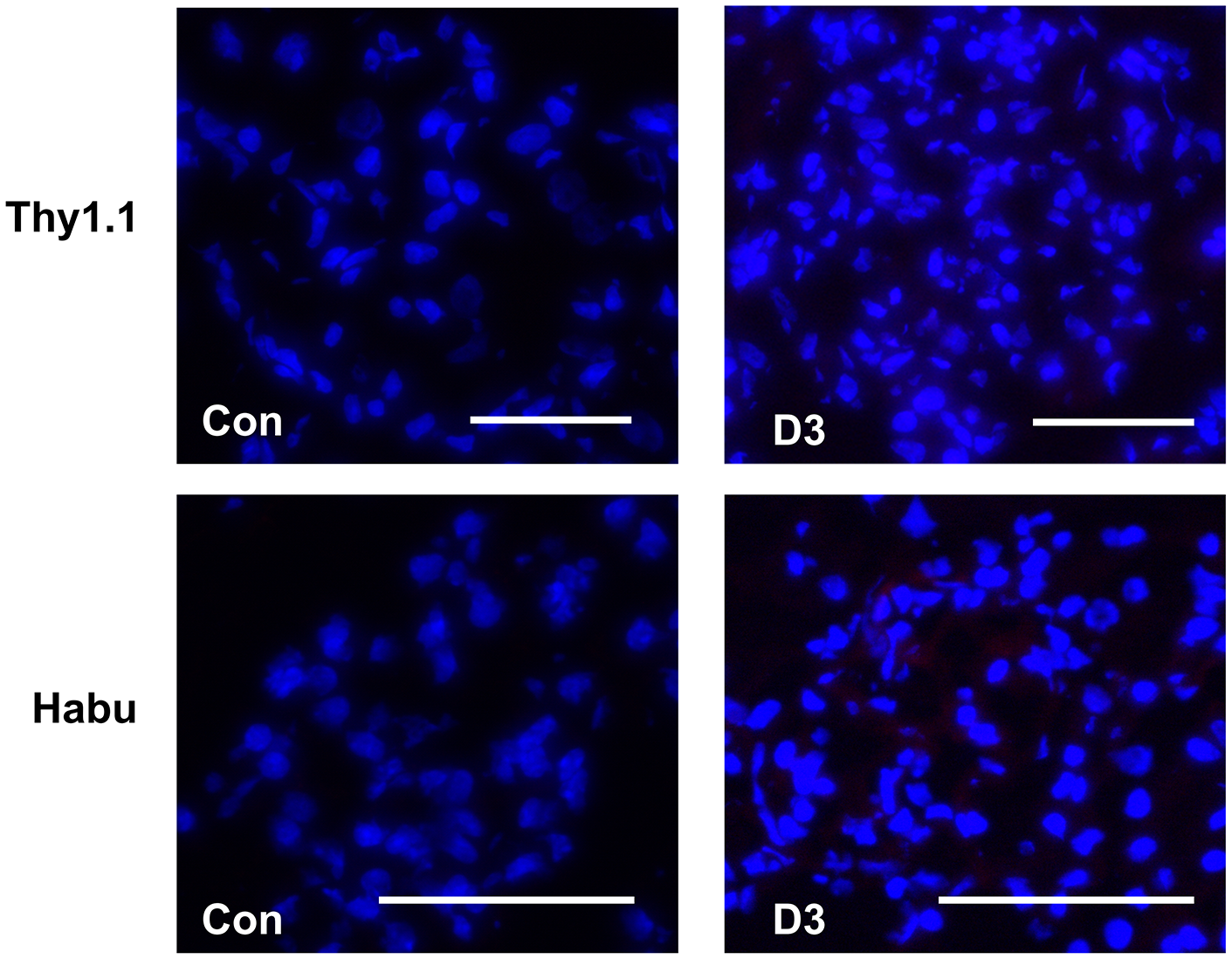

Supplement: Figure S2 — An IgG isotype control antibody was used on 4-μm-thick kidney sections to determine MHC class II expression in Thy1.1 and Habu GN glomeruli via immunofluorescence. Original magnification, × 400, Scale bar = 50 μm. [file Image_2.TIF]

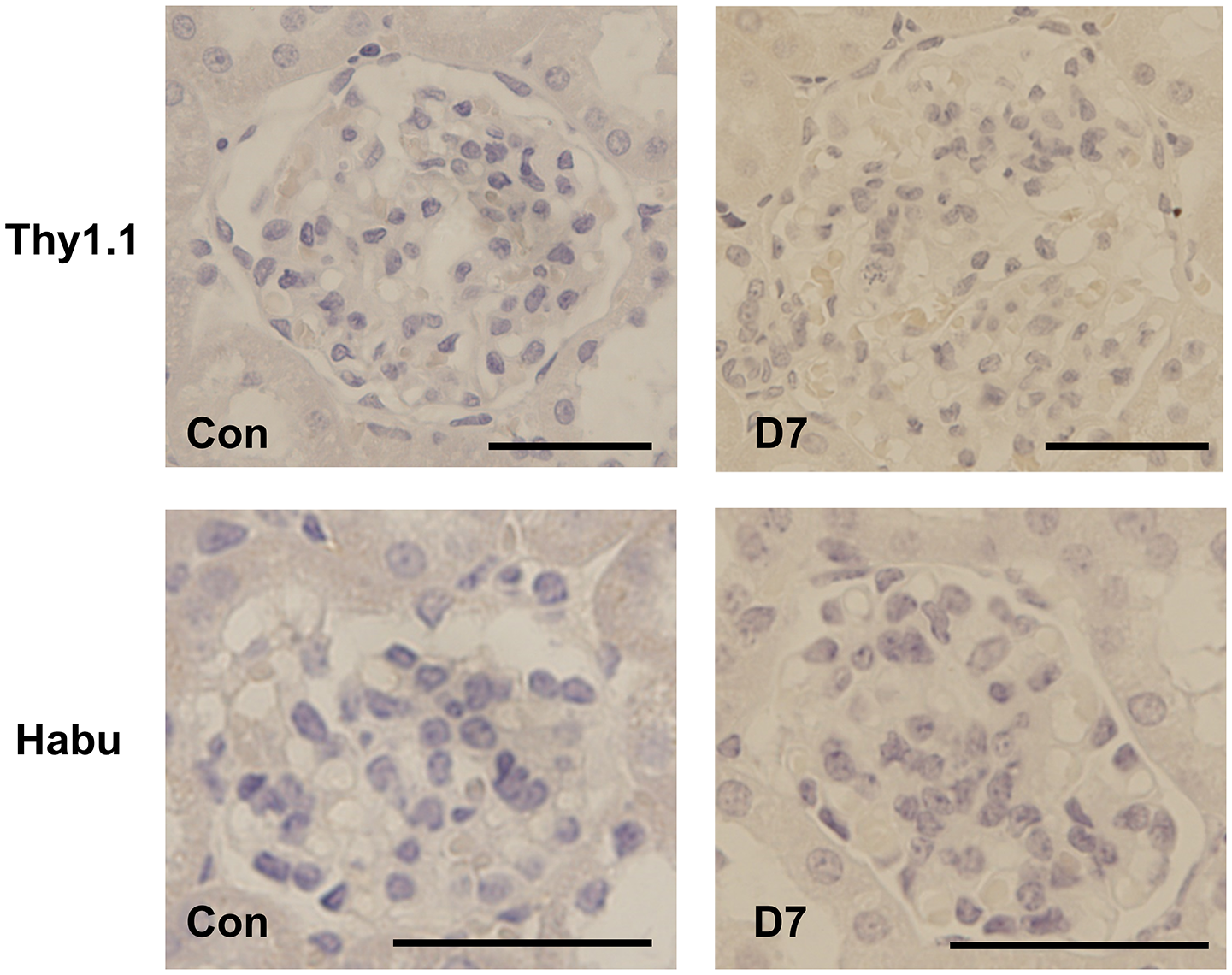

Supplement: Figure S3 — An IgG isotype control antibody was used on kidney sections to determine P-STAT1 in Thy1.1 expression in Habu GN glomeruli via immunohistochemistry. Original magnification, × 400, Scale bar = 50 μm. [file Image_3.TIF]

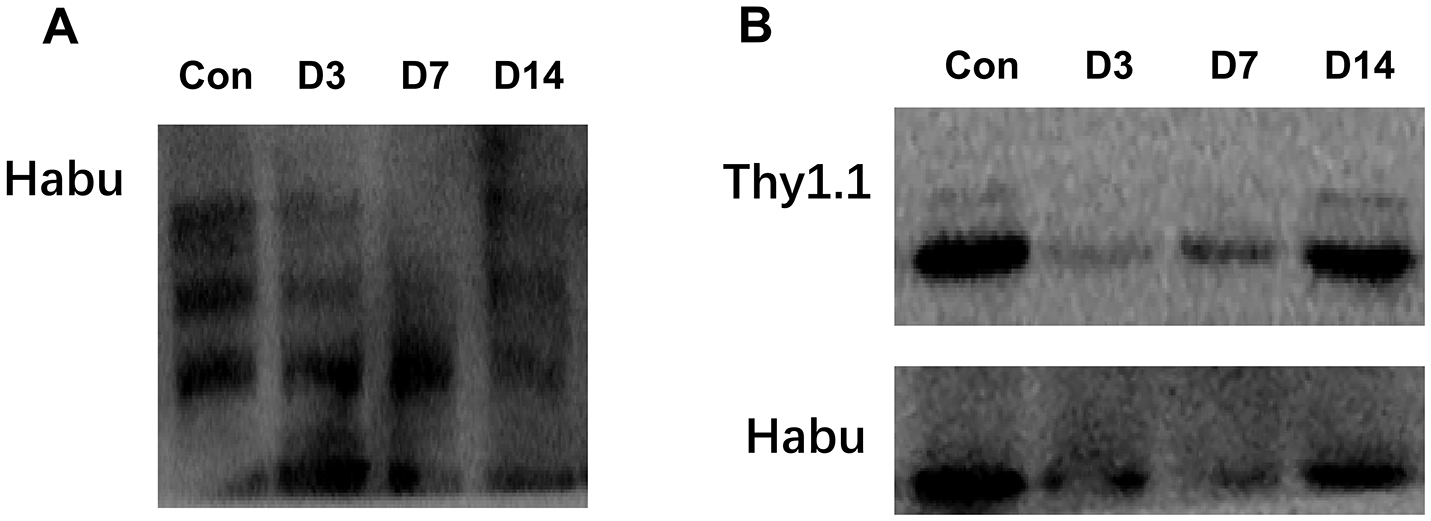

Supplement: Figure S4 — SOCS1 protein expression was detected in glomeruli of MsGN models. (A) SOCS1 protein expression was detected in glomeruli of Habu models using an SOCS1 antibody from Santa Cruz Biotechnology. (B) SOCS1 protein expression was detected in glomeruli of Thy1.1 and Habu models using an SOCS1 antibody from Abcam. [file Image_4.TIF]
