# Supplementary material for: Suppressor of Cytokine Signaling-1/STAT1 Regulates Renal Inflammation in Mesangial Proliferative Glomerulonephritis Models
Source: Front Immunol. 2018 Aug 30;9:1982. doi: 10.3389/fimmu.2018.01982 (PMC6125399; doi:10.3389/fimmu.2018.01982)
Supplement: Supplementary Table 1 — List of primers used in reverse transcriptase–polymerase chain reaction. [file Table_1.DOCX]

**Supplementary table**

List of primers used in reverse transcriptase–polymerase chain reaction

| Name | Sense primer (5'–3') | Antisense primer (5'–3') |
| --- | --- | --- |
| Rat *IFN-γ* | ACAACCCACAGATCCAGC | TCAGCACCGACTCCTTTT |
| Rat *TNF-α* | CCACGCTCTTCTGTCTACTG | GGGAACTTCTCCTCCTTGTT |
| Rat *IL-12A* | GAATCACAGCGGCGAGAC | TGAAGGCATGGAGCAGGA |
| Rat *IL-12B* | GCTGGTGTCTCCACTCAT | AGTCCACCTCTACAACATAAA |
| Rat *IL-6* | GGAGTTCCGTTTCTACCT | CTCTGGCTTTGTCTTTCT |
| Rat *IL-17A* | AGAGGGAGCCTGAGAAGT | GGCGGACAATAGAGGAAA |
| Rat *IL-23A* | CCAGCAGTGGCAGCGTTCT | TGCTCCGTGGGCAAAGACC |
| Rat *MHC class II* | AAACCCTCCTCCCAGAAA | CAGACCCACAAACAACCC |
| Rat *SOCS1* | CCTGAACTCCACGCCTACC | TGTGCAAAGATACTGGGAACAT |
| Rat *GAPDH* | ACAAGATGGTGAAGGTCGGTG | AGAAGGCAGCCCTGGTAACC |
| Mus *IFN-γ* | CAACAACATAAGCGTCAT | TCAAACTTGGCAATACTC |
| Mus *TNF-α* | AGCAGAAGCTCCCTCAGCGAGGACA | TGTCCTCGCTGAGGGAGCTTCTGCT |
| Mus *IL-12A* | GAATCATAATGGCGAGAC | TTCACTCTGTAAGGGTCTG |
| Mus *IL-12B* | CCCATTCCTACTTCTCCC | ACGCACCTTTCTGGTTACAC |
| Mus *IL-6* | GGACCAAGACCATCCAAT | GCTTAGGCATAACGCACT |
| Mus *IL-17A* | CTACCTCAACCGTTCCAC | GAGCTTCCCAGATCACAG |
| Mus *IL-23A* | CTGAGAAGCAGGGAACAA | GCAACAGCCATAGCATTA |
| Rat *MHC class II* | CACCCTCATCTGCTTTGT | CTGCGACTGACTTGCTAT |
| Mus *SOCS1* | TTCCGCTCCCACTCCGATTA | TAGAAGCCGCAGGCGTCCAG |
| Mus *GAPDH* | GGCAAATTCAACGGCACAGT | AGATGGTGATGGGCTTCCC |
| Mus *CIITA* | TCTGGACTGGGAAACCTC | CCATAATGCCATTGTATCACT |
| Mus *IP-10* | AAGCTATGTGGAGGTGCG | TAGGGAGGACAAGGAGGG |
| Mus *Mig* | TAACTTGATCCCATCTTCA | TACAGCCACAACCTTCTA |
